# A case of congenital *plasmodium vivax* malaria from a temperate region in central china

**DOI:** 10.1186/1475-2875-11-182

**Published:** 2012-06-06

**Authors:** Xue Liu, Zhi-Yong Tao, Qiang Fang, Xue-Mei Wang, Hui Zhang, Jose A Stoute, Hui Xia, Liwang Cui

**Affiliations:** 1 Bengbu First People’s Hospital, 229 Tu Shan Road, Bengbu, 233010, P. R. of China; 2 Department of Microbiology and Parasitology, Bengbu Medical College, 2600 Donghai Dadao, Bengbu, 233030, P.R. of China; 3 Department of Medicine, Pennsylvania State University College of Medicine, 500 University Drive, MC H036, Room C6860, Hershey, PA, 17033, USA; 4 Department of Entomology, Pennsylvania State University, 537 ASI Building, University Park, PA, 16802, USA

## Abstract

In February 2011, a rare case of congenital *Plasmodium vivax* malaria was diagnosed in a temperate region of Central China. An infant developed intermittent fever 20 days after delivery. Since this occurred during the non-transmission winter season in a low malaria endemic region and the infant’s mother did not have a clear malaria history or showed malaria symptoms at the time of the delivery, malaria infection was not suspected at the beginning. Later, on suspicion of potential malignant haematological illness due to persistence of the fever, bone marrow smear was examined, which revealed infection by *P. vivax* parasite. This rare case of congenital vivax malaria underlines that malaria diagnosis might need to be included in the healthcare of neonates born in vivax-endemic areas.

## Background

Congenital malaria is associated with significant neonatal mortality [1]. Some studies performed in hyperendemic areas suggested that congenital malaria is uncommon [2-5], whereas others have shown that it occurs significantly more frequently than was previously considered [6-9]. In comparison, reports of congenital malaria in hypoendemic areas have been even rarer. Since the symptoms of congenital malaria are usually atypical, and difficult to diagnose, it often results in delayed anti-malarial treatment [10]. Therefore, it was suggested that malaria diagnosis should be included as part of routine healthcare for all neonates in malaria hyperendemic areas [9,11].

Compared to *Plasmodium falciparumPlasmodium vivax* has a much wider distribution outside Africa and it extends far into the temperate zones. Recently, *P. vivax* has been shown to be not as benign as it was previously thought and is associated with complications, such as severe anaemia, respiratory distress, malnutrition, and even coma [12]. In addition, *P. vivax* has been found to be a major cause of morbidity in young children [13]. Besides, *P. vivax* can cause relapses due to the presence of long-lived latent forms in the liver, known as hypnozoites. Without radical treatment to remove the hypnozoites, patients may suffer relapses. In the tropics, *P. vivax* strains are characterized by early primary infection followed by frequent relapses. In temperate areas, however, primary infection tends to occur later with long intervals and fewer and later relapses as adaptation to climatic conditions to avoid the lengthy winter when the mosquito vector is unavailable [14,15]. Relapse not only renders *P. vivax* resistant to eradication, but also makes diagnosis difficult during the winter season when natural malaria transmission is absent. Here is a report of a rare case of congenital vivax malaria, which occurred in a hypoendemic, temperate area during the cold winter season.

## Case presentation

A 20-year old primigravida delivered a boy on February 7, 2011, in the Department Obstetrics of Bengbu First People's Hospital, Anhui Province, China. The newborn had a gestational age of 37 weeks, weighed 1.95 kg, and the Apgar score was 10 at 1 minute. Because of low body weight compared with normal birth weight of 3.6 ± 0.4 kg at the same gestational age, he was transferred to the neonatal intensive care unit immediately after delivery. He was discharged from the hospital at the age of 20 days. At home, he had fever for five hours on the same day, and was returned and admitted to the hospital for treatment. Physical and laboratory examinations were performed immediately.

Physical examination revealed that the infant had a body temperature of 38.5°C and body weight of 1.97 Kg. His liver was palpable with a span of 2 cm both below the right costal margin and below the sternum, while the spleen was not palpable. Coarse breath sounds without rales were heard over both lung fields. There was no evidence of jaundice. Laboratory blood test results showed that the infant had a white blood cell (WBC) count of 34.4 × 10^9^/L with 39.5% polymorphonuclear leukocytes and 52.2% lymphocytes, and a platelet (PLT) count of 57 × 10^9^/L. His haemoglobin (Hb) level was 147 g/L. He had a total bilirubin level of 295.6 μmol/L (≤205 μmol/L as normal) and direct bilirubin level of 16.5 μmol/L (≤34 μmol/L as normal). Blood cultures were performed and the result was negative.

**Figure 1:**
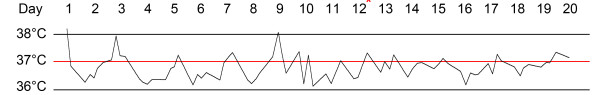
**The infant’s body temperature.** Shown here is an intermittent fever pattern prior to the initiation of CQ treatment on the 12^th^ day anti-malarial(shown as an asterisk) and subsequent resolution of the fever.

**Figure 2:**
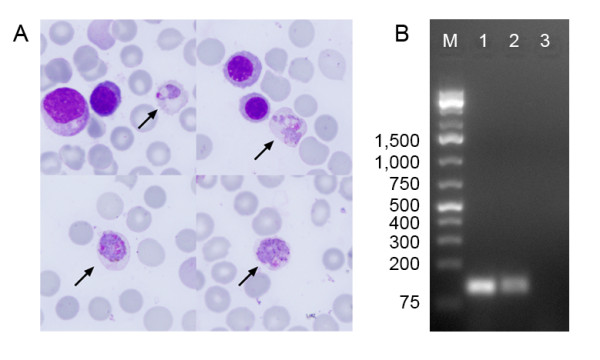
**Diagnosis of the congenital malaria.** (A) Giemsa-stained film of the bone marrow aspiration from the infants showing *P. vivax* infected erythrocytes (arrows, magnification 1,000×). (B) Confirmation of the diagnosis by nested PCR showing the ~125 bp PCR band specific for *P. vivax*. M, DNA Ladder, Lane 1, *P. vivax* positive control; Lane 2, DNA extracted from the infant’s bone marrow film; Lane 3, Negative control.

The admission diagnosis was upper respiratory tract infection associated with low body weight. The infant received intravenous injections of the antibiotic cefoperazone (0.1 g, twice daily) for 12 days. Meanwhile, his axillary temperature was monitored every four hours. Despite the treatment, he still showed an intermittent fever (Figure 1). Blood routine tests were ordered on the 2^nd^, 4^th^, 8^th^, 12^th^, and 19^th^ day since admission. On the 8^th^ day of antibiotic treatment, his WBC count returned to the normal range, but his Hb and PLT count continuously decreased (Table 1). A malignant haematological illness was suspected, and a bone marrow aspiration was conducted. Surprisingly, examination of the bone marrow smear revealed *P. vivax* parasitized erythrocytes (Figure 2A). This finding was later verified by nested PCR using DNA isolated from the blood film (Figure 2B) [16].

**Table 1:** Results of blood routine tests of the infant

Day*	WBC (×10^9^/L)	Hb (g/L)	RBC (×10^12^/L)	Hematocrit (%)	PLT (×10^9^/L)
2	34.4	147	4.32	54.0	57
4	12.7	142	4.55	47.3	46
8	8.6	87	2.72	17.1	36
12	7.3	78	2.45	24.5	141
19	8.0	98	3.23	32.5	138

*Day since the infant was readmitted to the hospital. Anti-malarial treatment started on day 12

The infant was diagnosed as having congenital malaria caused by *P. vivax*, a rare event in this area. Antibiotics were immediately discontinued and anti-malarial treatment was begun. He was given oral chloroquine phosphate (CQ) at 25 mg/kg for three days. Since parasites were not completely cleared on day 3, additional three-day treatment at 12 mg/kg was given. Since the initiation of CQ treatment, he became afebrile (Figure 1). One week later, his Hb level rose to 98 g/L (Table 1). He became aparasitaemic seven days after CQ treatment was started. He was discharged on the same day.

During the anti-malarial treatment of the infant, his mother also received examinations for malaria infection. Two blood smears obtained at one week’s interval were both microscopically negative. She was not given anti-malarial treatment. In retrospect, the infant’s mother recalled that she had an intermittent fever for about a week in the 6^th^ month of her pregnancy, but the symptoms were resolved without anti-malarial treatment. Two follow-up visits at 6 and 12 months showed that both the infant and mother did not develop further malaria symptoms during this period.

## Conclusions

In recent years, *P. vivax* has become the predominant malaria parasite species in China [17]. Especially in the temperate central provinces where malaria transmission is unstable, *P. vivax* was the only species responsible for malaria outbreaks occurred recently [18]. With considerable control efforts, the annual malaria incidence rate in Anhui Province was significantly reduced in recent years [17]. In city, *P. vivax* malaria is rare, especially during the winter when malaria transmission is interrupted. The *P. vivax* parasite in this region is typical of temperate strains with a long relapse pattern. Occasionally, patients develop malaria during the non-transmission season, which often makes proper malaria diagnosis difficult. Febrile patients during winter time are normally not considered for diagnosis of malaria infection. In the present case, the patient was a newborn, and the onset of malaria symptoms occurred when he was 20 days old. Since the average temperature in February in Bengbu city was 3.8°C, natural transmission of malaria could not have occurred. In addition, his mother was a local resident without a travel history outside of Anhui province during her pregnancy. During her pregnancy, she did not receive anti-malarial treatment. Therefore, it was impossible to connect the infant’s fever to malaria infection at the beginning. Only when haematological examination was performed on suspicion of haematological illness, he was diagnosed of having vivax malaria infection. Since natural transmission was extremely unlikely, the only possibility was transmission of the parasite from the mother to the foetus during her pregnancy or at the time of delivery.

Malaria infection during pregnancy can have a huge impact on both the mother and the foetus. It can lead to still birth, premature delivery, and low birth weight. Evidently, congenital malaria is another serious challenge for neonates’ healthcare [1,11]. For babies born to mothers with active blood stage malaria infection or history of malaria infection during pregnancy, aggressive monitoring of congenital malaria should be adopted. Currently, there is little information about the prevalence of congenital *P. vivax* malaria [19], and equally unknown is the impact of congenital malaria on neonates. Therefore, this case of congenital vivax malaria during a non-transmission season in a temperate region cautions that neonates born in malaria-endemic regions with fever should be examined for congenital malaria, especially when anemia or thrombocytopenia occurs.

Since primaquine is contraindicated in pregnancy, there is no radical malaria treatment strategy for pregnant women with absolute safety to the foetus. Although it has been shown that weekly CQ prophylaxis against vivax malaria in pregnant women is safe and effective in preventing vivax malaria [20], chemoprophylaxis for pregnant women living under risk of *P. vivax* has not been widely used [11]. In regions, such as the central provinces of China, where clinical CQ resistance in *P. vivax* malaria has not been documented and *in vitro* assays showed that *P. vivax* was sensitive to CQ [21], CQ prophylaxis against vivax malaria during pregnancy might be offered to high-risk populations. In addition, considering the effects of congenital malaria on the foetus and infant [1,13], antenatal care in malaria endemic areas to detect and treat malaria episodes during pregnancy needs to be actively implemented.

## Competing interests

The authors declare that they have no competing interests.

## Authors’ contributions

XL, ZYT drafted the manuscript. XL, ZYT and QF contributed expertise in the laboratory diagnosis and specie identification. XMW, HZ carried out data collection and epidemiological study. HX conceived this case report. HX, LC and JS contributed to case analysis. All authors have read and approved the final manuscript.
